# Enabling UAV Navigation with Sensor and Environmental Uncertainty in Cluttered and GPS-Denied Environments

**DOI:** 10.3390/s16050666

**Published:** 2016-05-10

**Authors:** Fernando Vanegas, Felipe Gonzalez

**Affiliations:** Australian Research Centre for Aerospace Automation (ARCAA), Queensland University of Technology (QUT), 2 George St, Brisbane, QLD 4000, Australia; felipe.gonzalez@qut.edu.au

**Keywords:** unmanned aircraft, UAV target detection, Partially-Observable Markov Decision Process (POMDP), path planning, Robotic Operating System (ROS), uncertainty, robust navigation

## Abstract

Unmanned Aerial Vehicles (UAV) can navigate with low risk in obstacle-free environments using ground control stations that plan a series of GPS waypoints as a path to follow. This GPS waypoint navigation does however become dangerous in environments where the GPS signal is faulty or is only present in some places and when the airspace is filled with obstacles. UAV navigation then becomes challenging because the UAV uses other sensors, which in turn generate uncertainty about its localisation and motion systems, especially if the UAV is a low cost platform. Additional uncertainty affects the mission when the UAV goal location is only partially known and can only be discovered by exploring and detecting a target. This navigation problem is established in this research as a Partially-Observable Markov Decision Process (POMDP), so as to produce a policy that maps a set of motion commands to belief states and observations. The policy is calculated and updated on-line while flying with a newly-developed system for UAV Uncertainty-Based Navigation (UBNAV), to navigate in cluttered and GPS-denied environments using observations and executing motion commands instead of waypoints. Experimental results in both simulation and real flight tests show that the UAV finds a path on-line to a region where it can explore and detect a target without colliding with obstacles. UBNAV provides a new method and an enabling technology for scientists to implement and test UAV navigation missions with uncertainty where targets must be detected using on-line POMDP in real flight scenarios.

## 1. Introduction

Ground, underwater and aerial robots are widely used for environmental monitoring and target detection missions [1,2,3,4,5]. Among these robots, UAVs use ground control stations to plan a path to a goal before flying, using Global Positioning System (GPS) sensors as their source of localisation [6,7,8,9,10]. However, reliable GPS localisation is not always available due to occlusions or the absence of the satellite signal. The accuracy of such localisation systems also decreases for low cost UAVs. Flying in GPS-denied environments and with only on-board sensors as the source of localisation is challenging, particularly when there are obstacles in the airspace that need to be avoided or if there is uncertainty in the goal location, which requires the UAV to fly and explore the area until the target is detected.

POMDP is a mathematical framework to model decision making problems with different sources of uncertainty [11,12,13,14], which makes it useful for implementing UAV navigation in cluttered and GPS-denied environments. The performance of POMDP solvers has improved, especially in the ability to cope with larger numbers of states |S| > 2000 and observations |O| > 100 [15,16], which is the case in UAV navigation missions. UAV navigation has been formulated previously as a Partially-Observable Markov Decision Process (POMDP) [17,18,19]. However, most of these studies only consider simulated scenarios [20] and use off-line solvers to compute the policy [21]. Another research study [22] modelled the uncertainty in target localisation using a belie state and planned a series of actions to solve the mission; however, this study relied on accurate localisation of the UAV in its environment.

In a POMDP, it is desirable to calculate a large number of possible sequences of actions and observations in order to increase the quality of the planning, but these must be done within a limited time, which depends on the system dynamics. This paper presents a system in which the actions that the UAV executes are designed based on the capabilities of the motion control system. This approach provides a method that enables us to model the POMDP transition function using characteristic step responses of four states in the UAV, and it also allows us to define a maximum computation time for each iteration of the on-line POMDP algorithm.

We developed *UAV Uncertainty-Based Navigation* (UBNAV) for UAVs to navigate in GPS-denied environments by executing a policy that takes into account different sources of uncertainty. This policy is calculated on-line by a POMDP path planning algorithm. Two on-line POMDP solvers, *Partially-Observable Monte Carlo Planning* (POMCP) [14] and *Adaptive Belief Tree* (ABT) [16], will be compared and integrated into a modular system architecture along with a motion control module and a perception module. The system uses a low cost multi-rotor UAV flying in a 3D indoor space with no GPS signal available. UBNAV updates its motion plan on-line while flying based on sensor observations taking into account motion and localisation uncertainties in both the UAV and the target.

UBNAV incorporates different sources of uncertainty into a UAV motion plan on-line in order to navigate challenging scenarios using only on-board sensors. The system uses motion commands instead of waypoints and updates a policy after receiving feedback from a perception module. This approach provides ease in modelling the decoupled system dynamics by using time step responses for a set of holonomic actions in four states of the UAV. The system also guides the UAV towards regions where it can localise better in order to reduce the uncertainty in localisation of both the UAV and the goal target. UBNAV enables researchers to implement and flight test on-line POMDP algorithms for the purpose of UAV navigation in GPS-denied environments or where perception has high degrees of uncertainty.

## 2. UAV Navigation as a Sequential Decision Problem

### 2.1. Markov Decision Processes and Partially-Observable Markov Decision Processes

A Markov Decision Process (MDP) is an effective mathematical framework to model sequential decision problems affected by uncertainties [23]. When MDP is used for UAV or robotic missions, the objective is to generate a policy that allows the UAV or robot to decide what sequence of actions it should take, taking into account the uncertainties in motion, in order to maximise a return or cost function.

MDPs assume that the robot states are completely observable. However, a UAV or robot that has limitations in perception due to its sensors and the environment in which it is moving has partial observability of its states. The perception of the UAV or robot is not completely accurate or is insufficient, which means there are deviations from the real state, creating partial observability.

Partially-Observable Markov Decision Processes (POMDP) incorporate the uncertainties in sensors and the partial observability of the UAV and target locations in the environment [24]. Formally, a POMDP consists of the following elements (S,A,O,T,Z,R,γ). *S* represents the set of states in the environment; *A* stands for the set of actions the UAV or robot can execute; *O* is the set of observations; *T* is the transition function for the state after taking an action; *Z* is the distribution function describing the probability of observing *o* from state *s* after taking action *a*; *R* is the set of rewards for every state; and *γ* is the discount factor. POMDP relies on the concept of belief, *b*, or belief state, which is a probability distribution of the system over all of the possible states in its state-space representation at a particular time.

POMDP enables us to represent the observations that a UAV or robot receives o∈O using observation functions *Z* that map probability distributions to states and actions. A policy π:B→A allocates an action *a* to each belief b∈B, which is the set of possible beliefs. Given the current belief *b*, the objective of a POMDP algorithm is to find an optimal policy that maximizes an accumulated discounted return when following a sequence of actions suggested by the policy *π*. The accumulated *discounted return* $R_{t}$ is the sum of the discounted rewards after executing every action in the sequence from time *t* onwards $R_{t}=\sum_{k=t}^{\infty }\gamma^{k-t}r_{k}$, where $r_{k}$ is the immediate reward received at particular time step *t* for taking action $a_{t}$, and *γ* is a discount factor that models the importance of actions in the future. Choosing a value lower than one for *γ* signifies that actions performed in short-term steps are more important than those in long-term steps. The *value function* $V^{\pi }$ is the expected return from belief *b* when following policy *π*, $V^{\pi }\left(b\right)=E\left[\sum_{t=0}^{\infty }\gamma^{k-t}r_{k}|b,\pi \right]$. An optimal policy for solving the POMDP problem maximizes the value function $\pi^{*}\left(b\right)={arg max}_{\pi }V^{\pi }\left(b\right)$.

Uncertainty in UAV localisation can be represented using an observation function *Z*. This observation function models the UAV localisation according to the accuracy of the GPS signal in the scenario and the precision of the onboard odometry system. The objectives of the UAV mission can be represented using a reward function *R*, and the motion plan is the calculated policy $\pi^{*}$ that maximises an expected discounted accumulated return for a sequence of motion commands or actions. The UAV dynamics and the motion uncertainty are incorporated into the POMDP using the transition function *T*, which enables one to predict the next state $s^{\prime }$ of the UAV after an action *a* is executed.

### 2.2. POMCP and ABT

In this study, we implemented and tested two of the fastest on-line POMDP algorithms (to the authors knowledge), *Partially-Observable Monte Carlo Planning* (POMCP) [14] and *Adaptive Belief Tree* (ABT) [16], in hardware and software in order to test the system for UAV navigation missions.

POMCP [14] uses the Monte Carlo Tree Search (MCTS) algorithm [25] to produce a search tree of possible subsequent belief states *b* and proved to be successful in problems with large domains. The algorithm reduces the search domain by concentrating only on the states that can be reached by the UAV or robot from its initial belief state $b_{0}$ after performing actions and receiving observations. In order to calculate the transition to the next states, the algorithm uses a black box simulator that has the transition functions (T) for the UAV. In our case, the UAV dynamics are the transition function for the UAV and are modelled as motion equations in four degrees of freedom using a continuous state space. The POMCP algorithm samples states from an initial belief state and performs thousands of Monte Carlo simulations applying the set of actions that the UAV can perform; see Table 1. POMCP uses the MCTS algorithm to guide the search of the sequences of actions in the reachable belief state space and builds a search tree according to the observations received after the UAV performs the actions.

POMCP initially runs a planning stage, where it generates a policy *π* that is stored as a tree with nodes containing belie states that are represented by particles. The algorithm then outputs the action *a* that maximises an expected accumulated return. Afterwards, this action *a* is executed, and then, an observation *o* is received. With this observation, POMCP performs an update of the belief state *b* by updating the tree and selects the node that matches the observation received in the tree search. The algorithm incorporates new particles to avoid particle deprivation and initiates a new search round from the matched node. The search depth of the planning stage is controlled by the planning horizon *h* parameter. The longer the planning horizon, the more time the algorithm will take to build the search tree.

ABT is an on-line POMDP solver that also uses Monte Carlo simulations to predict future belief states and a set of state particles to represent the belief state [16]. Both algorithms, POMCP and ABT, generate a search tree to store the policy. The root of the tree is a node containing the state particles representing the initial belief of the environment. The tree branches out according to the probability of selecting actions and receiving observations.

ABT updates the policy *π* after receiving an observation *o* by keeping the search tree without deleting it, which increases the number of possible sampling states in the tree search. On the other hand, POMCP only keeps the belief node that matches the received observation. The rest of the search tree is deleted, and a new round of calculations for building the policy takes place after every iteration.

## 3. System Architecture

**Figure 1:**
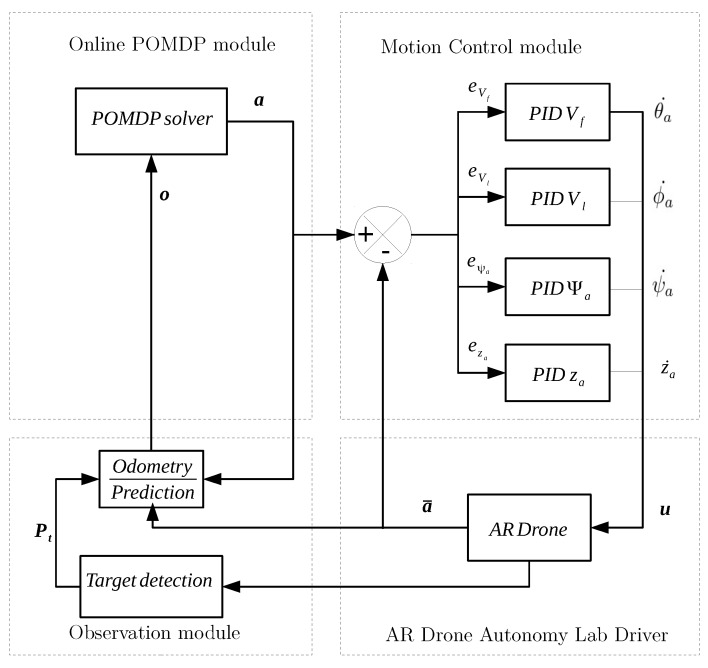
POMDP ROS system architecture.

UBNAV is developed as a modular system that consists of four modules that interact with a multi-copter UAV. A diagram with the system architecture is shown in Figure 1. The system contains an on-line POMDP module that runs the POMDP on-line solver and outputs actions that are executed by the motion control module. It also includes a formulation of the navigation mission as a POMDP. The on-line POMDP module updates the belief state *b* according to received observations o. We use existing open-source code for the POMCP [14] and ABT algorithms [26] as the base code and modified the codes to integrate all of the modules using the Robotic Operating System (ROS) [27]. The motion control module controls four states in the UAV using PID controllers. Actions in the formulated POMDP are a combination of reference values for each of the four controllers. The observation module uses the on-board sensors information and calculates and updates the UAV position. The final module is the AR Drone Autonomy Lab driver for ROS [28], which reads multiple UAV sensors and receives commands to control the roll, pitch, yaw and thrust of the UAV.

All modules execute in parallel in different threads with different update rates. The motion control module, the observation module and the AR Drone driver module execute at 100 Hz in order to control the dynamics of the UAV and to compute the odometry and estimate the location of the UAV. On the other hand, the POMDP solver executes at 1 Hz in order to guarantee that the UAV reaches a steady state after executing actions and to have a policy that enables the UAV to accomplish the mission. The system source code is written in C++, is available as ROS packages and can be provided as open-source code upon request.

### 3.1. On-Line POMDP Module

We did several modifications and additions to the POMDP solvers’ source code in order to allow them to work as ROS nodes and to integrate them into the modular system. We also created a visualisation scenario in order to examine the performance of the solvers in simulation.

The on-line POMDP module runs the on-line POMDP planning algorithms. This module initialises the parameters for planning horizon *h*, the map of the environment, target location, odometry uncertainty and an initial belief state $b_{0}$. This module produces a policy *π* based on the initial belie state $b_{0}$ and outputs the action *a* to be executed according to the calculated policy. The action *a* is then executed by the motion control module, and the observation module calculates the UAV position using the on-board sensed velocity, altitude and heading angle. The observation module also checks whether the target is detected by the downward-looking camera and detects obstacles located within approximately 1 m in front of the UAV with an on-board front-looking camera.

Once the observation is received, the POMDP node updates the belie state *b*, to match the received observation and replenishing particles until a time-out is reached. Based on the current belie state *b*, the POMDP solver calculates a new policy *π* (POMCP) or updates the policy *π* (ABT) and outputs the subsequent action *a* based on the updated policy *π*.

### 3.2. Motion Control Module

The UAV motion control module is composed of four independent controllers that actuate on the following states of the UAV: Forward velocity $V_{f}$, lateral velocity $V_{l}$, yaw angle (heading angle) $\Psi_{a}$ and altitude $z_{a}$. The motion control module receives reference states $a=\{V_{f}^{*},V_{l}^{*},\Psi_{a}^{*},z_{a}^{*}\}$ from the POMDP solver (see Table 1) and subtracts the actual states $\bar{a}=\left\{V_{f},V_{l},\Psi_{a},z_{a}\right\}$ from the references sates a to generate error signals that are used by each of the PID controllers.

The output of the PIDs is a control vector $u=\{\dot{\theta_{a}},\dot{\phi_{a}},\dot{\psi_{a}},\dot{z_{a}}\}$, where $\dot{\theta_{a}}$, $\dot{\phi_{a}}$ and $\dot{\psi_{a}}$ are pitch, roll and yaw rates, respectively, and $\dot{z_{a}}$ is the rate of climb. These outputs are sent to the AR Drone Autonomy lab driver, which transforms them into control signals that are sent to the UAV.

The UAV on-board navigation system calculates forward $V_{f}$ and lateral $V_{l}$ velocities using optical flow obtained from the downward-looking camera. The UAV obtains the yaw angle $\Psi_{a}$ from the IMU and magnetometer readings and the altitude $z_{a}$ from on-board ultrasonic and barometric pressure sensors that are fused using a proprietary Kalman filter.

**Figure 2:**
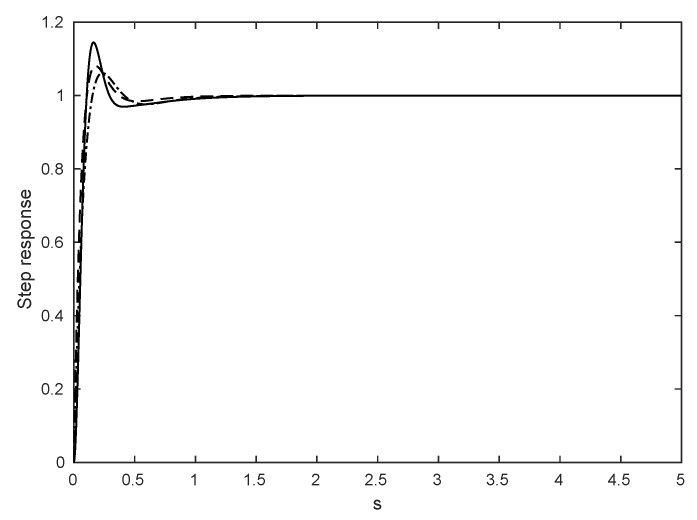
PID time responses to a unit step input for $V_{f}$ and $V_{l}$ (dashed line), Ψ (dash-dot line) and *z* (solid line). All PID controllers reach steady state within 1 s.

The duration of an action execution $T_{a}$ is chosen to guarantee that the PID controller reaches a steady state when transitioning from different actions for all states, as shown in Figure 2. Therefore, we set the POMDP solver time to to be equal to the action duration, that is $T_{P}=T_{a}$.

### 3.3. Observation Module

The observation module calculates the current multi-rotor position and heading angle based on the sensed forward and lateral velocities, accelerations and the measured yaw angle $\Psi_{a}$. The forward and lateral velocities in the UAV’s frame are transformed to the fixed world frame and are integrated to calculate the UAV coordinates $x_{a}$ and $y_{a}$ in the world frame. The UAV altitude $z_{a}$ is also read from the AR Drone ROS driver and is calculated on-board.

The UAV position $P_{a}=\left\{x_{a},y_{a},z_{a},\Psi_{a}\right\}$ is calculated based on the actual states $\bar{a}$ obtained from the on-board sensors from the AR Drone ROS driver. The POMDP solver calculates and updates the policy based on the position and heading angle that the UAV will have by the time the previous action is finished, *i.e.*, $t-T_{a}$. Thus, a prediction of the UAV position and heading angle $P_{a_{t+1}}$ is calculated based on the current UAV position and yaw angle in the world frame, the action currently being executed and the action duration time $T_{a}$. This prediction is calculated using the characteristic step responses for the four degrees of freedom shown in Figure 2, the commanded state reference values a and the actual state values $\bar{a}$.

The observation module also detects and calculates the target position $P_{t}$ by using a specific tag figure and the AR Drone Autonomy Lab driver to determine whether the tag is present in the downward looking camera image and its position within the image. The last type of observation in the module identifies obstacles. In this case, we use Augmented Reality (AR) tags that are placed on the obstacles and which can be detected with the front camera using the ROS package Arsys for reading Aruco-type tags [29].

The use of the AR tags enables the system to have a global source of localisation since the tags can give an accurate indication of the UAV camera pose with respect to the world frame by using coordinate frame transformations. However, these tags can only be detected when the AR tag is located within the UAV front camera Field Of View (FOV) and within a distance of approximately 1 m. The model of the downward looking camera FOV is described in Section 4.5.

## 4. Navigation and Target Detection

### 4.1. Problem Description and Formulation

**Figure 3:**
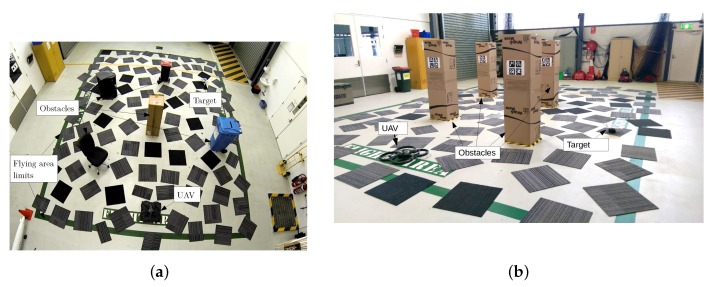
Two typical scenarios for the navigation and target detection problem. (**a**) Scenario with five obstacles; (**b**) scenario with four obstacles with Augmented Reality (AR) tags.

Consider a multi-rotor UAV flying in a 3D space in a GPS-denied environment filled with obstacles. An example of such an environment is the indoor scenario shown in Figure 3. The UAV has the mission to navigate within a limited region of the airspace with boundaries $x_{lim}$, $y_{lim}$ and $z_{lim}$ in the *x*, *y* and *z* coordinates, respectively. The UAV’s task is to fly from the initial hovering position to a region where the target is believed to be located in order to explore and detect it using an onboard downward-looking camera, avoiding obstacles whose location is known by the UAV navigation system. After taking off, the UAV hovers for five seconds before starting the mission. This initial hovering incorporates uncertainty into the UAV position due to the UAV drifting around the take off position. The target is stationary, and its location is known by the system with uncertainty.

The navigation and target detection must be done using only on-board sensors, whilst overcoming uncertainties in the UAV states information and uncertainty in target location. We use continuous state representation to model the transition function and a discrete set of observations *O* as described in Section 4.5.

The problem is formulated as a POMDP with the following elements: the state of the aircraft in the environment (S), the set of actions that the multi-rotor can execute (A), the transition function describing the state transition after applying a specific action (T), the observation model (O) that represents the sensed state and uncertainty of the UAV after taking an action and the reward function (R).

### 4.2. State Variables (*S*)

The state variables considered in the POMDP formulation are the position and yaw (heading) angle of the UAV in the world frame $P_{a}=\left(x_{a},y_{a},z_{a},\Psi_{a}\right)$, the target’s position $P_{t}=\left(x_{t},y_{t},z_{t}\right)$ and the UAV’s forward $V_{f}$ and lateral velocity $V_{l}$.

### 4.3. Actions (*A*)

There are seven possible actions a∈A that the UAV can execute: an action to go forward at speed $V_{f}$, an action to go backwards at speed $-V_{f}$, an action to roll left at speed $V_{l}$ and an action to roll right at speed $-V_{l}$. Actions up and down increase or decrease the altitude by 0.3 m, with UAV’s forward $V_{f}$ =0 m/s and lateral velocity $V_{l}$ = 0 m/s. The hover action maintains the UAV’s velocity at zero. The set of actions is summarised in Table 1.

### 4.4. Transition Functions (*T*)

In order to represent the system dynamics, a kinematic model is described as in Equation (2) to Equation (3). The position of the aircraft is determined by calculating the change in position due to its current velocity, which is controlled by the motion control module and is selected according to the commanded action. A transformation from the UAV’s frame to the world frame is also calculated in these equations.

A normal probability distribution with mean value and standard deviation <1 m around the take off position, as shown in Equation (1), is used to model this uncertainty on the UAV initial position.

The orientation of the UAV is determined by its heading angle $\Psi_{a}$. The uncertainty in motion is incorporated in the system by adding a small deviation to the heading angle $\sigma_{a}$ using a normal distribution with mean value equal to the desired heading angle and restricted to the range $-2.0^{\circ }\;<\;\sigma_{a}\;<\;2.0^{\circ }$, which represents the uncertainty on the yaw angle control system.

A discrete table with the characteristic values of a step input response was obtained experimentally for each of the controllers for every degree of freedom in the motion controller module. These step responses (see Figure 2) are included in the transition function in order to incorporate the transient changes in the UAV speed when it transitions from action to action after receiving the command from the on-line planner. The uncertainty in UAV position due to the initial hovering is modelled as a Gaussian probability distribution, described by Equation (1), with mean value *μ* and standard deviation *σ* < 1 m.

(1) P(x)=1σ2πe-x-μ2-x-μ22σ22σ2

(2) $$\begin{array}{c} \left[\begin{array}{c} \Delta x_{a_{t}}\\ \Delta y_{a_{t}}\\ \Delta z_{a_{t}} \end{array}\right]=\left[\begin{array}{ccc} \cos(\Psi_{a_{t}}+\sigma_{a_{t}}) & -\sin(\Psi_{a_{t}}+\sigma_{a_{t}}) & 0\\ \sin(\Psi_{a_{t}}+\sigma_{a_{t}}) & \cos(\Psi_{a_{t}}+\sigma_{a_{t}}) & 0\\ 0 & 0 & 1 \end{array}\right]\left[\begin{array}{c} V_{a_{f_{t}}}\Delta t\\ V_{a_{l_{t}}}\Delta t\\ \Delta z_{a} \end{array}\right] \end{array}$$

(3) $$\left[\begin{array}{c} x_{a_{t+1}}\\ y_{a_{t+1}}\\ z_{a_{t+1}} \end{array}\right]=\left[\begin{array}{c} x_{a_{t}}\\ y_{a_{t}}\\ z_{a_{t}} \end{array}\right]+\left[\begin{array}{c} \Delta x_{a_{t}}\\ \Delta y_{a_{t}}\\ \Delta z_{a_{t}} \end{array}\right]$$

where $x_{a_{t}}$, $y_{a_{t}}$ and $z_{a_{t}}$ are the *x*, *y* and *z* aircraft coordinates at time *t*, $V_{a_{f_{t}}}$ and $V_{a_{l_{t}}}$ are forward and lateral velocities in the multi-rotor’s frame at time *t* and $\Psi_{a_{t}}$ and $\sigma_{a_{t}}$ are heading and heading deviation at time *t*.

### 4.5. Observation Model (*O*)

An observation for the POMDP model is composed of: (1) the UAV position in the world frame with onboard odometry as the source; (2) the UAV position with reduced uncertainty in the world frame if obstacles are detected; and (3) the target location if it is detected by the downward-looking camera. The UAV odometry calculation has an uncertainty that is approximated using a Gaussian distribution, as in Equation (1). The mean value of this distribution is the UAV position calculated by the odometry system, and the variance is equal to the error in the odometry calculation. This error is bigger for UAV positions calculated based only on optical flow sensor and smaller if the AR tags are detected in any of the obstacles by the front camera.

Equation (4) is used to calculate the $x_{a}$ and $y_{a}$ positions of the UAV.

(4) $$\left[\begin{array}{c} x_{a_{t+1}}\\ y_{a_{t+1}} \end{array}\right]=\left[\begin{array}{c} x_{a_{t}}\\ y_{a_{t}} \end{array}\right]+\left[\begin{array}{cc} \cos(\Psi_{a_{t}}) & -\sin(\Psi_{a_{t}})\\ \sin(\Psi_{a_{t}}) & \cos(\Psi_{a_{t}}) \end{array}\right]\left[\begin{array}{c} V_{a_{f_{t}}}\\ V_{a_{l_{t}}} \end{array}\right]\Delta t$$

If the target is detected by the onboard downward-looking camera, the AR Drone ROS driver provides the target position within the image. This position is transformed to a position in the world frame.

**Figure 4:**
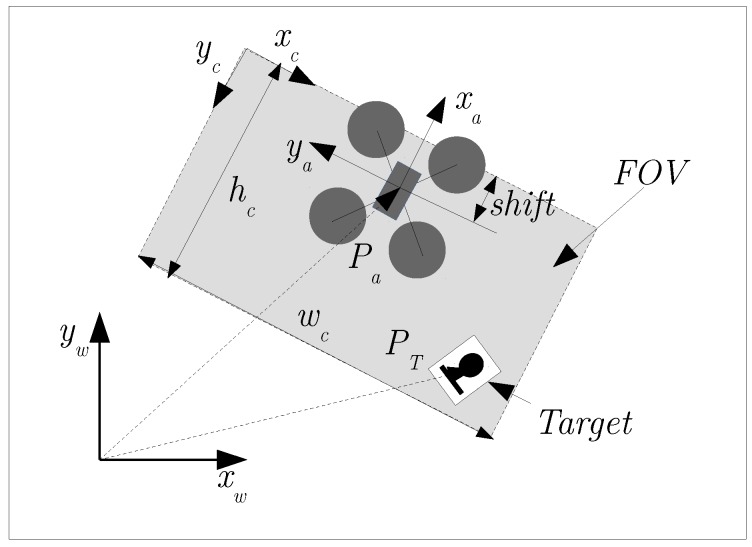
Field of view parameters for the UAV onboard camera, target and world frames.

The FOV of the downward-looking camera, shown in Figure 4, is modelled and defined experimentally as follows: the image width depends on the UAV altitude and is defined as $w_{c}=z_{a}\alpha_{w}$; the image height is defined as $h_{c}=z_{a}\alpha_{h}$; and there is a shift from the UAV frame to camera frame that is defined as $shift=z_{a}\alpha_{s}$, where $\alpha_{h}=0.56$, $\alpha_{w}=0.96$ and $\alpha_{s}=0.2256$ are intrinsic parameters of the camera and are obtained by camera calibration and experimental tests.

### 4.6. Reward Function (*R*)

The objectives of the UAV navigation mission can be represented in the POMDP using a reward function. The UAV receives a high reward of 300 if it detects the target within the downward-looking camera FOV. Hitting an obstacle or going out of the scenario incurs a penalty of −70, and every other movement carries a cost of −2. The values of the reward and penalties were selected based on existing test cases of POMDP problems. These values were tuned by experimentation on a large number of simulations (≈500) and flight tests (≈50). A summary of the reward function values is shown in Table 2.

### 4.7. Discount Factor (*γ*)

A discount factor *γ* of 0.97 was selected by experimentation on a large number of simulations (≈500) and flight tests (≈50) and taking into account the distance travelled by the UAV in every step at the selected speed and the size of the flying area.

## 5. Results and Discussion

We conducted simulation and real flight experiments for four tests cases to compare the performance of ABT and POMCP.

### 5.1. Simulation

We tested the UAV navigation formulation in simulation with both solvers, POMCP and ABT, by running each algorithm, using the model dynamics presented in Section 4.4 and Section 4.5. We used boost C++ library random generators in order to have high quality normal distributions as described in Section 4.1.

**Figure 5:**
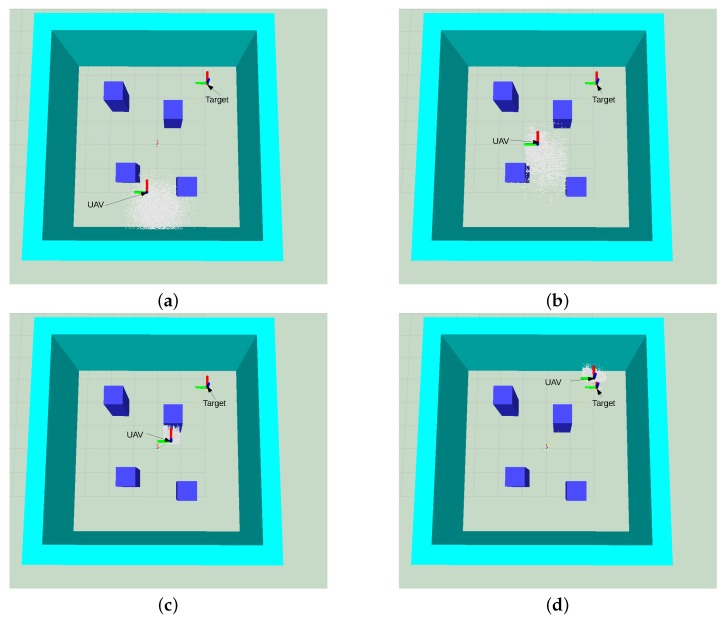
Belief evolution for the navigation problem using the POMCP solver. Target’s position (red); UAV position (white). (**a**) Initial belief, UAV location (white particles) and target location (red particles); (**b**) belief after some steps; (**c**) UAV in front of obstacle; uncertainty is reduced; (**d**) target within UAV downward camera FOV.

The target was placed at four different locations inside the flying area, and the UAV was initially located around (3.0,0.55,0.7), with uncertainty in each of the locations, as described by Equation (1). Each target location was tested in simulation by running POMCP and ABT 100 times. We created a simulated 3D model of the environment to visualise the UAV path in the scenario to inspect the evolution of the belief state of the system and for visualising the UAV position and the target location as clouds of white and red particles, respectively (Figure 5). Initially, the UAV position, represented by the white particle cloud in Figure 5a, has an uncertainty around the starting position at the bottom of the image. The spread of the particles represents the drift caused by UAV take off and initial hovering. This uncertainty increases as the UAV flies towards the goal (Figure 5b). As the UAV gets closer to one of the obstacles and detects the AR tag attached to the obstacle with the front camera, the uncertainty in the UAV position is reduced, as seen in Figure 5c. The UAV then flies towards the target until it detects it, shown in Figure 5d, and the uncertainty in its position is reduced by the knowledge that it acquires from the position of the target in the image taken by the downward-looking camera.

A summary of the results for the discounted return and the time to detect the target for each test in simulation and in real flight is shown in Table 3.

**Figure 6:**
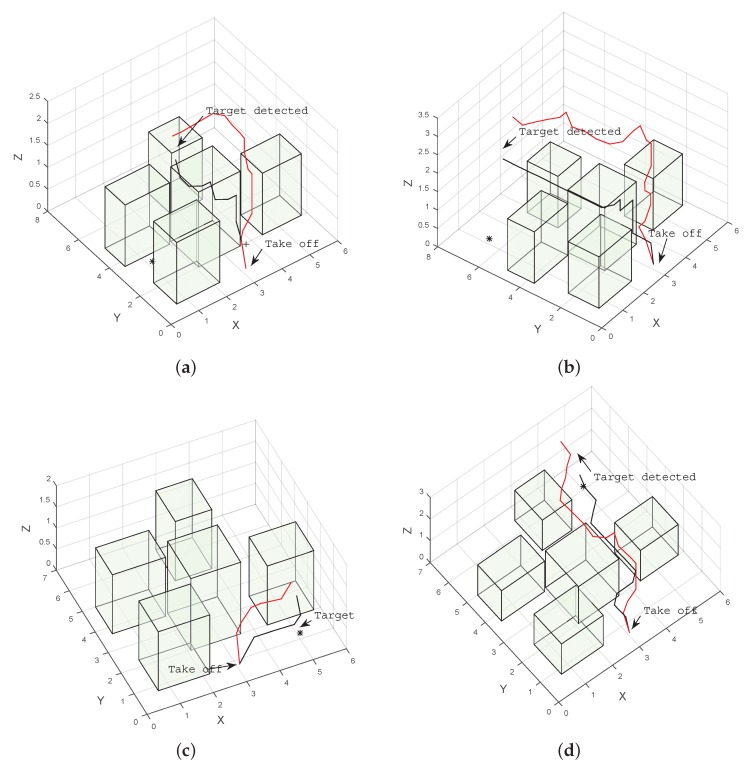
Example of trajectories to four different target locations produced by ABT (black), and POMCP (red) in simulation. (**a**) Target located at (1.0, 3.0, 0.15); (**b**) target located at (1.0, 6.5, 0.15); (**c**) target located at (5.0, 1.0, 0.15); (**d**) target located at (5.0, 6.0, 0.15).

**Figure 7:**
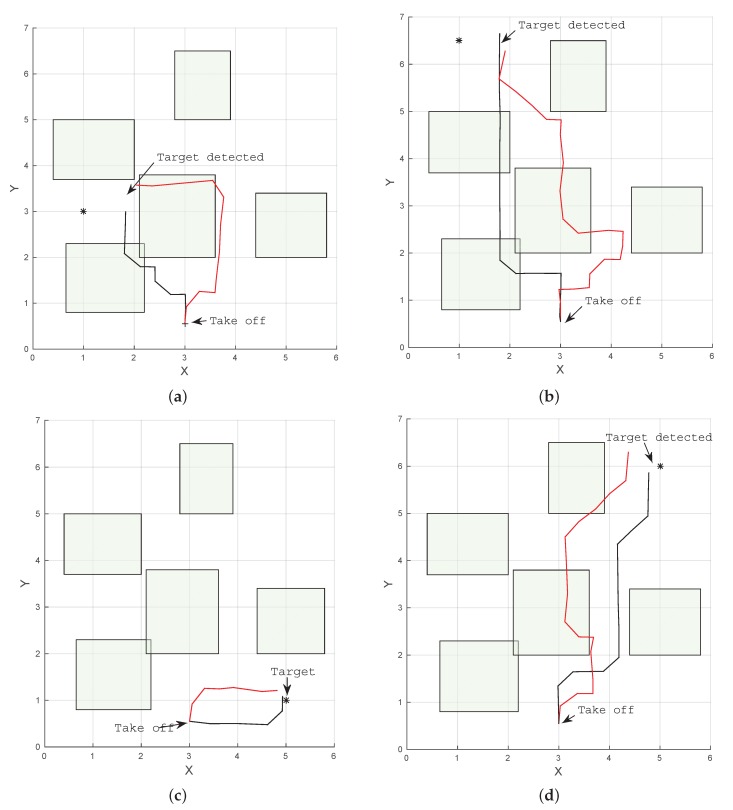
Example of trajectories to four different target locations produced by ABT (black) and POMCP (red) in simulation. (**a**) Target located at (1.0, 3.0, 0.15); (**b**) target located at (1.0, 6.5, 0.15); (**c**) target located at (5.0, 1.0, 0.15); (**d**) target located at (5.0, 6.0, 0.15).

The results indicate that for all cases in the simulation, both POMDP solvers were able to reach and detect the target in less than 20 s. Results also show that paths produced by ABT are shorter than the ones produced by POMCP (Figure 6 and Figure 7). This can also be seen in the flight time to detect the target, which is shorter for ABT in three of the four cases; see Table 3. In the case of the target located at (5,6,0.15), the path computed by ABT shows that the UAV takes a trajectory flying between the obstacles, whereas with POMCP, the UAV flies over the obstacles to avoid the risk of collision.

### 5.2. Real Flight Tests

We conducted experiments in real flight scenarios with the target located at different positions. We ran the system 10 times for each location of the target for both solvers to compare their performance. We also compare two scenarios, one with five obstacles and another with four taller obstacles with AR tags on each of the obstacles, which enable the UAV to correct the uncertainty in the onboard odometry once the tags are in the FOV of the front camera.

The UAV has to take off from an initial position and hover for 4 s to initialise its on-board sensors. The forward (pitch) and lateral (roll) velocity controllers start to actuate when taking off, and the altitude controller sets an initial value for the UAV to hover at 0.7 m above the ground.

The initial POMDP navigation policy is computed once the UAV is airborne, and the POMDP module outputs an action after 4 s of hovering. The system computes a new policy at each step of 1 s of duration.

**Figure 8:**
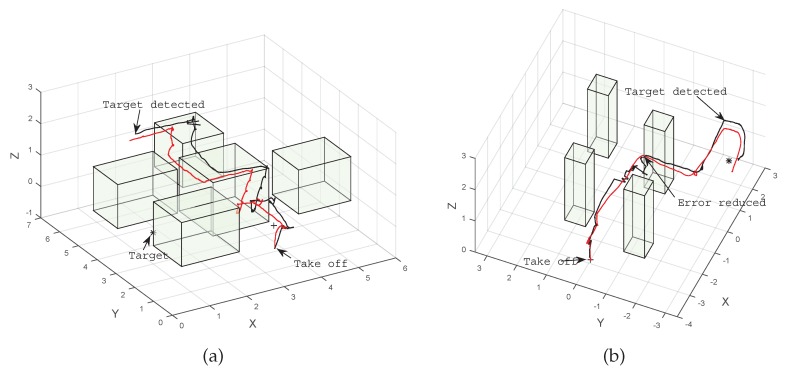
Comparison of onboard computed position (red) *vs.* the VICON positioning system (black). (**a**) Without resetting position error; (**b**) resetting position error with AR tags.

Figure 8a shows a comparison of the UAV position estimation performed by the observation node and using the VICON positioning system. Even though there is an error in the estimated path, the system is robust enough to account for this drift, is able to find a path using the UAV on-board odometry and finds the target without colliding with the obstacles.

The system was also tested incorporating the detection of the obstacles by the UAV, using AR tags that allow for a more reliable source of positioning in places where the UAV is closer to the obstacles than the onboard odometry. A comparison of the UAV position estimation against the VICON system using the AR tags to reduce the uncertainty is shown in Figure 8b. Results indicate that the system is able to reduce the uncertainty and the error in the position estimation using AR tags as beacons in the environment.

**Figure 9:**
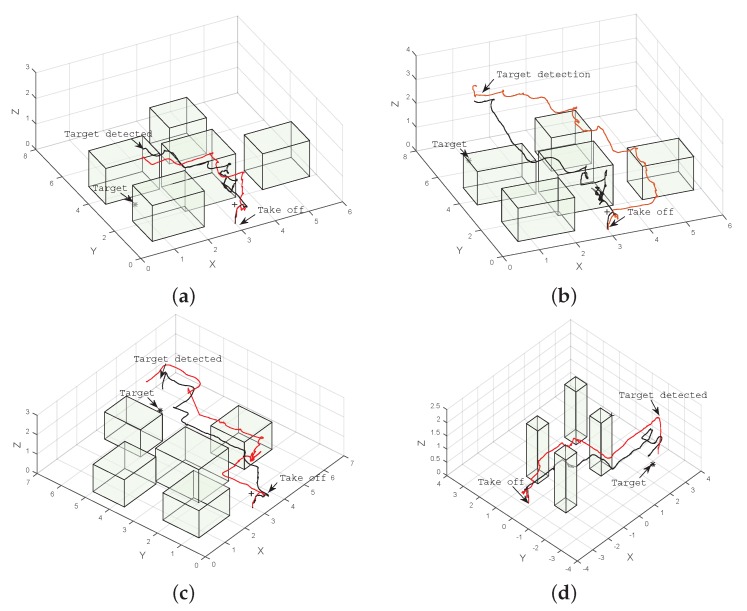
Example of trajectories to four different target locations produced by ABT (black) and POMCP (red). (**a**) Target located at (1.0, 3.0, 0.15); (**b**) target located at (1.0, 6.5, 0.15); (**c**) target located at (5.0, 6.0, 0.15); (**d**) target located at (2.65, −2.33, 0.15).

**Figure 10:**
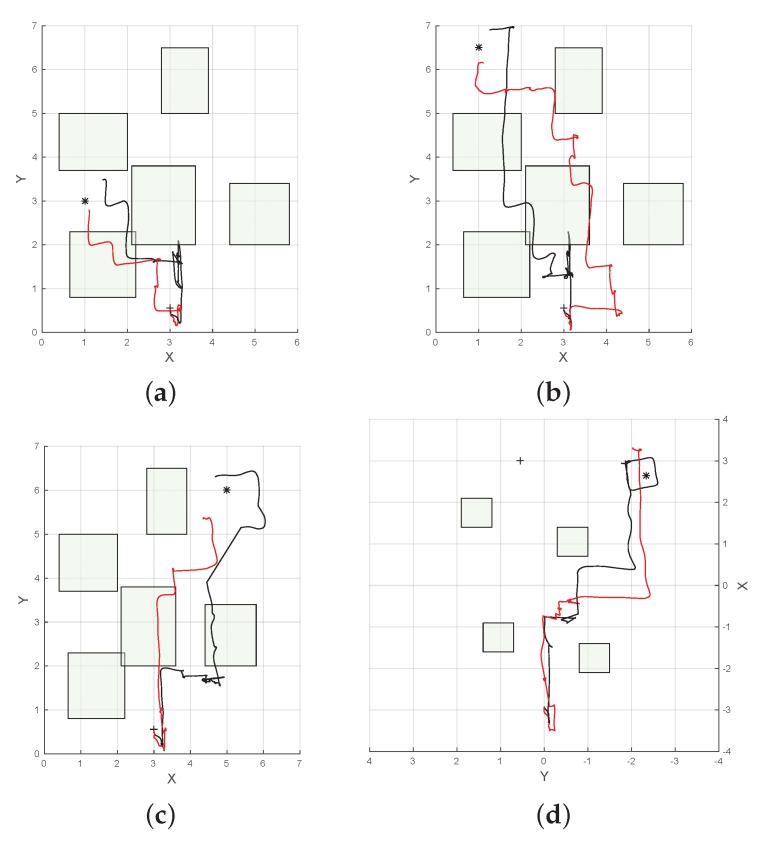
Example of trajectories to four different target locations produced by ABT (black) and POMCP (red) in simulation. (**a**) Target located at (1.0, 3.0, 0.15); (**b**) target located at (1.0, 6.5, 0.15); (**c**) target located at (5.0, 6.0, 0.15); (**d**) target located at (2.65, −2.33, 0.15).

Results in Table 3 indicate that the performance of both POMDP solvers is affected by real flight conditions, and the flight time to detect the target increases in all of the cases. The UAV can accomplish the mission in 100% of the cases using POMCP for both scenarios and 85% of the cases using ABT in real flight, as show in Table 3. A comparison of the paths flown by the UAV to four different target locations produced by POMCP and ABT algorithms are shown in Figure 9 and Figure 10.

Figure 9a–c show the trajectories taken by the UAV in a scenario with five obstacles. In this case, both solvers show that it is safer to fly over the obstacles to reach the target. On the other hand, Figure 9d shows that the algorithms find a safe route to the target by flying between obstacles in the scenario with four obstacles. However, in the last case, the UAV flies close to the obstacle first in order to reduce its uncertainty by detecting the beacon or landmark represented by the AR tag on the obstacle and then continues flying towards the target.

### 5.3. Recommendations for Implementation of UAV Navigation Using On-Line POMDP in a Real Flight

Implementation of UAV navigation in cluttered and GPS-denied environments formulated as a POMDP is a complex task. Some considerations should be taken into account for the implementation of the system for real flights.

The first consideration is to perform a thorough analysis of the dynamic capabilities of the UAV and, in our case, the design of motion controllers on four decoupled states. The design and tuning of these motion controllers takes time, repeated flight testing and varies according to UAV specifications. This step is indispensable and enables us to model the characteristic response of the UAV, which facilitates modelling the POMDP transition function based on the set of actions chosen.

The next consideration is to characterise the controllers’ time response by using system identification software and the data collected from the real flight tests. The transition function uses this characteristic time response to calculate the next state of the UAV after performing an action, and it is also used to simulate the performance of UBNAV. This step is also required for the implementation of navigation in other path planning algorithms, which require low level motion control that outputs a series of waypoints [3,4,10] or velocity commands that a lower level motion controller executes [30].

A third important consideration for a POMDP implementation is the observation function, which models the characteristics, ranges, accuracy, disturbances and noise measurements. Having an exact knowledge of all of these characteristics is not always possible, but some approximations can be made. If a camera is used as one of the sensors, then camera calibration and a model for the FOV that depends on the UAV altitude is needed. Several flight tests measuring the drift in the initial hovering and the yaw angle measurements are needed in order to approximate the model using Gaussian distributions.

A fourth consideration is the discount factor *γ* that is used to balance the importance of actions during the planning stage. Selecting a discount factor of one means that there is no discount applied to the return received after performing an action, which in turn gives the same importance to actions that are executed in the short term and in the long term. Conversely, assigning a value much lower than one discounts the return for actions executed in the long term and in turn gives more importance to short-term actions. In this study, the discount factor was selected by testing in 100 Monte Carlo simulations with values for the discount factor ranging from 0.95–1, taking into account that the UAV should not collide with obstacles (short-term goal) and should also reach and detect the target (long-term goal).

Finally, the reward function (R) was tuned by performing multiple Monte Carlo simulations. Values of the reward and penalties were tuned focusing first on reaching the primary goal, which is to detect the target. Afterwards, values of penalties for colliding with obstacles and going out of the flying area were tuned by fixing the reward for reaching the goal and running multiple simulations with different values for the penalty. Finally, in order for the UAV to choose shorter paths, values in the interval [0,20] for the motion penalty were tested running multiple simulations.

## 6. Conclusions

This study developed and tested UBNAV for UAV navigation in GPS-denied and cluttered environments where there is uncertainty in sensor perception and target localisation. UBNAV performed full navigation and target detecting missions using a low cost platform with only on-board sensors in GPS-denied environments and in the presence of obstacles. UBNAV allows researchers to implement, simulate and test different on-line POMDP algorithms for navigation with uncertainty and also permits the implementation of different motion controllers and perception modules into the system.

The system executes a set of holonomic actions instead of a classic waypoint approach. This approach facilitates the execution of the motion planning by modelling the UAV dynamics as a decoupled system with a set of actions that execute in four states of the UAV. It models the uncertainties in the motion and perception by including deviations in the states using Gaussian distributions.

The system also allows for selecting the planning time in an on-line POMDP solver for a UAV navigation mission, taking into account the duration of the actions. These actions are based on the time response of the motion controllers of the UAV.

Experimental results show that the system is robust enough to overcome uncertainties that are present during a flight mission in a GPS-denied and cluttered environment. UBNAV guides the UAV towards regions where it can localise better in order to reduce the uncertainty in the localisation of both the UAV and the goal target. Results indicate that the system successfully finds a path on-line for the UAV to navigate to a location where it could detect a target with its onboard downward-looking camera without colliding with obstacles for 100% of the time for the simulation and 96.25% of the time in 80 different real flight experiments.

The system has also the potential to be used in an outdoor environment with additional perception information by modelling a Global Positioning System (GPS) where the uncertainty can be introduced depending on the resolution and the quality of the GPS module. Adding the GPS module uncertainty and the wind disturbances as another source of uncertainty into the POMDP model along with real flight testing is currently being explored.

## Figures and Tables

**Table 1:** Summary of actions in navigation and target finding problem.

Action *a*	Forward Velocity $V_{f}^{*}$ (m/s)	Lateral Velocity $V_{l}^{*}$ (m/s)	Altitude Change $\Delta z_{a}^{*}$ (m)	Heading Angle $\Psi_{a}^{*}$ (deg)
Forward	0.6	0	0	90
Backward	-0.6	0	0	90
Roll left	0	0.6	0	90
Roll right	0	-0.6	0	90
Up	0	0	0.3	90
Down	0	0	-0.3	90
Hover	0	0	0	90

**Table 2:** Summary of rewards and costs in the UAV navigation problem.

Reward/Cost	Value
Detecting the target	300
Hitting an obstacle	-70
Out of region	-70
Movement	-2

**Table 3:** Simulation and real flight results for Adaptive Belief Tree (ABT) and POMCP.

Solver	Number of Obstacles in Scenario	Target Location (x, y, z)	Flight Time to Target (s) (Simulation)	Flight Time to Target (s) (Real Flight)	Success (No Collision) % (Real Flight)
POMCP	5	(5.0,6.0,0.15)	14	25	100
	4	(2.65,-2.33,0.15)	20	21	100
ABT	5	(5.0,6.0,0.15)	16	25	90
	4	(2.65,-2.33,0.15)	19	27	80
